# Quantitative MR in Paediatric Patients with Wilson Disease: A Case Series Review

**DOI:** 10.3390/children9050613

**Published:** 2022-04-25

**Authors:** Kamil Janowski, Elizabeth Shumbayawonda, Matt Kelly, Carlos Ferreira, Maciej Pronicki, Wieslawa Grajkowska, Magdalena Naorniakowska, Piotr Pawliszak, Sylwia Chełstowska, Elżbieta Jurkiewicz, Rajarshi Banerjee, Piotr Socha

**Affiliations:** 1 Department of Gastroenterology, Hepatology, Nutritional Disorders and Pediatrics, The Children’s Memorial Health Institute, 04-736 Warsaw, Poland; k.janowski@ipczd.pl (K.J.); m.naorniakowska@ipczd.pl (M.N.); p.socha@ipczd.pl (P.S.); 2 Perspectum Ltd., Oxford OX4 2LL, UK; matt.kelly@perspectum.com (M.K.); carlos.ferreira@perspectum.com (C.F.); rajarshi.banerjee@perspectum.com (R.B.); 3 Department of Pathology, The Children’s Memorial Health Institute, 04-736 Warsaw, Poland; m.pronicki@ipczd.pl (M.P.); w.grajkowska@ipczd.pl (W.G.); 4 Department of Diagnostic Imaging, The Children’s Memorial Health Institute, 04-736 Warsaw, Poland; p.pawliszak@ipczd.pl (P.P.); s.chelstowska@ipczd.pl (S.C.); e.jurkiewicz@ipczd.pl (E.J.)

**Keywords:** Wilson disease, multi-parametric MR, non-invasive, pediatric

## Abstract

Wilson disease (WD) is a liver disorder characterized by improper copper metabolism. Although non-invasive tools are currently used to support diagnosis and management, this is still an area of unmet need, as patients present with a wide range of symptoms. Our aim was to investigate the potential utility of multiparametric magnetic resonance imaging (mpMRI) and quantitative magnetic resonance cholangiopancreatography (MRCP+) to support patient management. MRI examinations of 7 children and young adults aged 8–16 years (six at diagnosis) were performed alongside a standard of care clinical and histological examination. Images were quantitatively analyzed to derive metrics of liver (corrected T1 (cT1; fibro-inflammation), MR liver fat (proton density fat fraction; PDFF)), and biliary health (MRCP+). MRI–PDFF provided a more dynamic characterization of fat compared with that provided by ultrasound. Those with elevated histological scores of fibrosis, inflammation, and steatosis had elevated mpMRI values. MRCP+ managed to identify dilatations in the biliary tree which were not observed during the standard of care examination. mpMRI and MRCP+ metrics show early promise as markers to assess both liver and biliary health in Wilson disease. Investigations to understand and explore the utility of these markers are warranted and should be performed.

## 1. Introduction

Wilson’s disease (WD) is a rare autosomal recessive disorder affecting the copper transport in hepatic cells, caused by mutations in the ATP7B gene on chromosome 13 [1,2]. Although the cause of the disease is fairly well understood, the diagnosis of WD still remains difficult due to a wide range of nonspecific symptoms including active hepatitis, cirrhosis, acute liver failure, asymptomatic liver enzyme elevations, and the absence of Kayser–Fleischer rings in up to 50% of patients [3,4,5]. As a result, although a liver biopsy is essential, diagnosis is not rendered on liver histology alone.

Non-invasive tools are already being used as assistive tools during diagnosis, evaluation, and monitoring of patients with WD. For instance, an ultrasound assessment using liver sonography has been used to investigate evidence of hepatitis, fibrosis, fatty infiltration, and cirrhosis [4]. MRI examinations have also demonstrated a good utility in detecting nodular infiltrations and liver contour abnormalities of cirrhosis in those with progressive hepatic dysfunction, and is a good marker of therapeutic response [6,7]; however, although MRI has shown great utility in characterising liver parenchyma, the paramagnetic influence of ionic copper has been identified as a cause of hypo-intensity in both T1- and T2-weighted images [6]. Multiparametric MRI (mpMRI) analyses combine both qualitative and quantitative MR image analysis to provide clinically useful information and can be used to support patient management [8]. For instance, corrected T1 (cT1; T1 corrected for the effects of paramagnetic metals such as iron (but not excluding copper)) has shown utility in disease characterisation [9,10,11,12], treatment response evaluation [13], clinical outcome prediction [14,15], and patient monitoring [16] in various liver diseases ranging from rare diseases such as autoimmune hepatitis [8,15,16,17], to more prevalent diseases such as non-alcoholic fatty liver disease (NAFLD) and non-alcoholic steatohepatitis (NASH) [18]. Moreover, when combined with MR liver fat (measured as proton density fat fraction; PDFF), cT1 has shown a better utility for identifying NASH than elastography [19] and standard liver function tests [18]. Together, cT1 and PDFF can also predict liver-related clinical events better than the NAFLD fibrosis score (NFS), enhanced liver fibrosis (ELF) test, and FIB-4 [20,21], and can predict all-cause mortality better than FibroScan^®^ and FIB-4 [21].

Most studies have focussed on the relationship between copper excretion and the severity of hepatic damage in WD when evaluating the biliary system [22,23,24,25]; however, very few studies have qualitatively or quantitatively evaluated the biliary structure of patients with this condition. Although the biliary tract is typically affected in primary sclerosing cholangitis (PSC), the differential diagnosis between PSC and WD can be a challenge. Magnetic resonance cholangiopancreatography (MRCP) has a recognised role in diagnosing and monitoring cholestatic diseases such as PSC [26]; nevertheless, similar to most qualitative image analyses, MRCP relies on subjective assessment, and hence, is affected by high inter-observer variability [27,28]. Quantitative MRCP (MRCP+) is a novel objective imaging tool that quantifies and produces a 3D model of the biliary tree [29]. Studies have shown the utility of MRCP+ in monitoring paediatric autoimmune liver diseases [17,30]. Moreover, MRCP+ metrics are comparable to ANALI scores and have been shown to improve risk-stratification in PSC [31]. MRCP could play a role in supporting the diagnosis of WD.

Due to its rarity, there is a scarcity of randomised controlled clinical trial data in WD which has resulted in variations in clinical guidelines concerning its diagnosis, treatment and monitoring [2]. For instance, whereas the guidance from the American Association for the Study of Liver Diseases (AASLD) [32] favours a more integrated algorithmic approach, combining clinical and biochemical parameters with liver biopsy, the clinical guidelines of the European Association for Study of Liver (EASL) [25] and ESPGHAN [33] seem to favour the use of non-invasive testing and low or no reliance on liver biopsy. Although liver histology is important for staging liver disease, it plays a smaller role in the management of patients; therefore, there is a growing need for more standardised patient management that depends less on expert opinion, and more on evidence-based guidance [2].

To date, no studies had investigated the use of quantitative mpMRI metrics to characterize hepatic disease burden in WD; therefore, in this series investigation, we evaluate the potential clinical utility of quantitative techniques to characterize the disease burden in WD. Our hypothesis was that the use of MRI techniques can potentially provide clinically useful information that is not currently provided by current imaging techniques used in clinical practice.

## 2. Materials and Methods

### 2.1. Patient Recruitment

WD patients were recruited in a prospective observational study (NCT03198104), looking at the utility of using multiparametric mpMRI in children and adolescents aged 6–18 with rare liver diseases (NCT03198104). WD was confirmed based on the Ferenci scoring system [34], as indicated by the clinical guidelines [25,33]. All WD patients included in this analysis had a diagnosis liver biopsy, and were under the care of hepatologists at the Children’s Memorial Health Institute in Warsaw (IPCZD). All patients underwent a research non-contrast MRI scan alongside their routine care assessments, including clinical history, physical and histological parameters, and serum liver biochemistry (Table 1). Six of the seven patients were recently diagnosed, had their mpMRI scan within three weeks post-diagnosis, and started treatment after their MRI scan. One patient was diagnosed 693 days (1.8 years) before being included in the study and was on zinc therapy (67.5 mg (3.2 mg/kg) of zinc daily).

### 2.2. Clinical, Laboratory and Histology Data

Percutaneous liver biopsy samples were assessed for Kleiner–Brunt fibrosis, lobular and portal inflammation, ballooning, steatosis, cholestasis, and necrosis by two experienced liver pathologists (as part of standard clinical care and as reported in [16]). In addition, for each patient, a diagnostic description was also recorded after physical examination at their consultation, where all patients received an ultrasound examination as part of their routine care examination (Table 2). All ultrasound examinations were performed by trained professionals at IPCZD.

### 2.3. Image Acquisition and Post-Processing: Multiparametric MRI and Quantitative MRCP

In each scan, non-contrast T1, T2*, and PDFF were acquired using the Liver*MultiScan*^®^ protocol (Perspectum Ltd., Oxford, UK). In a manner similar to that extensively described by Bachtiar and colleagues [35], four transverse slices were captured through the centre of the liver through the porta hepatis. Three circular regions of interest were placed on all four slices on the transverse T2* and PDFF maps during image analysis, whereas semi-automatic segmentation was used to delineate the images into whole liver segmentation maps to calculate cT1 [35].

In the same scanning session, 3D volumetric MRCP images were obtained from 72 continuous slices using 3D multi-shot fast/turbo spin-echo acquisitions. Parallel imaging techniques were used to decrease scanning time alongside fat suppression techniques. MRCP+ (Perspectum Ltd., Oxford, UK) was then used to extract and process the acquired MRCP data to produce a color-coded 3D model of the biliary tree [29].

**Figure 1:**
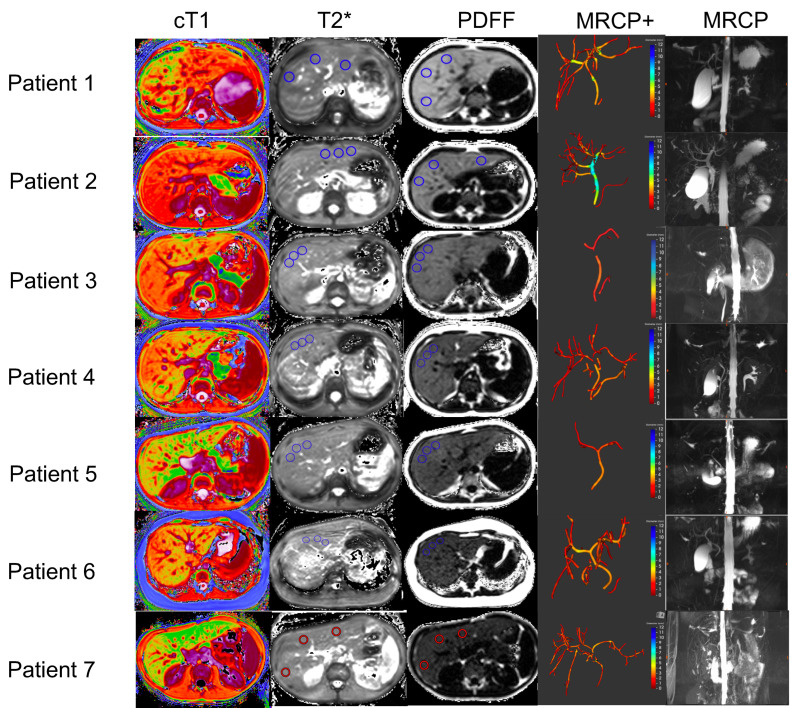
Representative biliary tree models, and cT1, T2*, and PDFF maps from all Wilson’s disease patients recruited in this study. In addition, the MRCP+ models of the biliary tree and the original MRCP images are also shown for each patient. In the cT1 maps, lower values (cooler colors) represent areas with lower cT1 values, and therefore, lower fibro−inflammation, whereas higher cT1 values (warmer colors) represent areas of the liver with higher fibro−inflammation. In the MRCP+ images, strictures are shown in red, whereas dilatations are shown in blue. cT1: corrected T1; PDFF: proton density fraction (MRI liver fat); magnetic resonance cholangiopancreatography (MRCP); quantitative magnetic resonance cholangiopancreatography (MRCP+).

All scans were performed at the IPCZD on 1.5 T Siemens Avanto systems (Siemens Healthineers, Erlangen, Germany) and the average scan duration was 20 min. All post-processing of the obtained images was performed by trained analysts who were blinded to all the clinical data. Figure 1 shows an illustration of a traditional MRCP and resultant quantitative models of the biliary tree derived using MRCP+, as well as the cT1, T2*, and PDFF maps for all WD patients. Inter-observer variability, as well as repeatability and reproducibility of these metrics, have been reported by McDonald et al. [11] McKay et al. [36] and Wilman et al. [37].

### 2.4. Statistical Analysis

Descriptive statistics were used to summarise participant characteristics. Continuous, normally distributed variables were reported as the mean and standard deviation (SD), whereas continuous, non-normally distributed variables were reported as the median and interquartile range (IQR), and categorical variables were reported as the frequency and percentage.

## 3. Results

### 3.1. Patient Demographics and Clinical Presentation

Kayser–Fleischer rings were present in three patients, and all seven patients had a Ferenci score of ≥6 [25,34]. The participants were aged 8–16 years (Table 1). Following laboratory testing, all patients had elevated hepatic copper content (>400 µg/g), 24 h urinary copper excretion, and the majority had low serum ceruloplasmin (<0.15 g/L). During a clinical examination using ultrasound imaging, all patients were confirmed to have a uniform liver structure, although one had an enlarged liver with hepatomegaly, fatty liver, and the presence of peritoneal fluid. All other patients were noted as having a slightly increased echogenicity of the liver using ultrasound imaging. None of the patients had comorbid autoimmune hepatitis, primary sclerosing cholangitis, diabetes (type 1 or 2), toxic liver failure, progressive familial intrahepatic cholestasis, or ascites (Table 2).

As shown in Table 1, the two oldest patients had a high fibrosis–collagen proportion (25%), and most patients had mild fibrosis (scores ≤ 2), lobular (scores ≤ 1) and portal inflammation (score = 1), as well as ballooning (scores ≤ 1). Most patients (4/7) had steatosis scores of 3, indicating severe steatosis (> 66% of the liver’s overall weight), whereas only two patients had prominent ballooning (score = 2). No patients in this study had cholestasis (in corroboration with initial clinical findings; Table 2); however, all patients had elevated alanine transaminase (ALT > ×2.5 upper limit of normal (ULN)) and aspartate transaminase (AST > ULN), with the majority of patients (6/7) having an international normalised ratio (INR) > 1. Despite having relatively similar histological scores, one patient had very elevated blood markers (AST and AST ≥ ×10 ULN; gamma-glutamyl transferase (GGT): 204 IU/L) and low total bilirubin (0.2 mg/dL). This patient did not have any significant findings on ultrasound imaging examination.

### 3.2. MR Imaging Findings

All patients in this study had elevated liver fat that was indicative of significant fatty liver disease (PDFF ≥ 10%), with those who had the lowest steatosis score (=2) having a PDFF of <16% (Table 3). Although findings obtained during the clinical workup using ultrasound imaging did not identify the presence of high liver fat, MRI–PDFF identified the presence of fatty liver infiltrations (as confirmed by histology findings). Although all patients had active fibro-inflammation (cT1 > 800 ms), the patient with hepatomegaly from physical examination had the highest cT1 (1017 ms).

On the ultrasound examination, no patients were found to have dilated bile ducts; however, MRCP+ detected at least four dilated ducts in the majority (5/7) of patients (Table 3). Furthermore, MRCP+ also showed that of the five patients who had dilatations in their biliary tree, four also had strictures. In addition, two newly diagnosed patients had an elevated (fasting) gallbladder volume > 60 mL.

## 4. Discussion

Multiparametric MR and MRCP+ are relatively new non-invasive diagnostic technologies, and to our knowledge, this is the first report evaluating their use in the context of Wilson’s disease. Thus, we investigated the potential usefulness of these techniques to characterise liver disease and provide clinically meaningful information regarding this orphan disease in a small sample of patients.

The rise in the utility of non-invasive technologies to support the diagnosis and management of various liver conditions is becoming more prominent. This has prompted clinical bodies such as EASL to update their clinical guidance [38] to specifically address the use of non-invasive technologies to evaluate liver disease severity and prognosis. Findings from this guideline update showed that MRI–PDFF is the most accurate non-invasive method for detecting and quantifying liver fat in patients with metabolic risk factors and/or suspected NAFLD, due to its ability to quantify its entire dynamic range [38]. This finding is significant as MRI–PDFF provides more detailed information about the patients’ liver health, as seen in this case series investigation where a steatosis score of 3 covered a PDFF range from 17–33%. This move to rely on MR-based technologies to support diagnosis has also been seen in PSC, where MRCP plays the leading role as a non-invasive technology in supporting diagnosis and disease monitoring [31].

Depending on the stage of the disease, zinc therapy and penicillamine have classically been used to treat patients with WD [25,33]; however, the assessment of treatment response and monitoring copper levels and urinary copper excretion is typically performed in combination with liver enzyme evaluation. These tests, although useful, neither fully characterize the degree of hepatic damage nor the amount of copper in the liver; therefore, in conjunction with the complex need for better and quicker diagnosis of the disease, improving patient management and monitoring is necessary. cT1 has been used for characterizing and monitoring NAFLD and NASH [18,19] and evaluating treatment response [13]. More recently, evidence has highlighted the utility of cT1 in managing and monitoring portal hypertension in adolescents and adults [14], as well as disease stratification, treatment monitoring, and identification of the sub-clinically active disease in children with autoimmune liver disease [16,17,30], using a cT1 of > 800 ms as a good indicator of active fibro-inflammation. All WD patients included in this early evaluation had active fibrosis and inflammation on histology and had a cT1 of > 800 ms. This early corroboration between imaging and histology is promising as it suggests the potential utility of this threshold in this disease (as reported and validated for other indicators).

A differential diagnosis of WD can be challenging as biliary diseases, such as PSC, may present similar symptoms, and some laboratory results may be similar, such as increased levels of copper in the liver [5,7]. MRCP+ has shown utility in monitoring and risk stratifying sclerosing cholangitis (primary and autoimmune) [17,30,31]. MRCP+ provides a quantitative overview of the worsening of the biliary tree, and thus provides beneficial information that can be used to support diagnosis and patient monitoring [39]. Interestingly, in this case series, five out of seven patients were identified as having dilatations, even though the evaluation of the following standard of care using ultrasound imaging did not identify any dilatations in the biliary tree. The assessment of the gallbladder forms part of the routine clinical and physical examination of WD, as patients can potentially develop cholecystitis. As part of its analysis, MRCP+ can characterise the gallbladder, and thus, it can be used to monitor gallbladder volume. For instance, in this case-series evaluation, 2 patients were identified as having gallbladder volumes > 60 mL, and thus, are ideal candidates for further clinical evaluation to rule out cholecystitis.

As non-invasive technologies begin to play a more prominent role in diagnosing and managing patients with liver disease, these quantitative markers should be evaluated in a bigger cohort to better understand their utility. For instance, although the use of MRI–PDFF (also validated by the Quantitative Imaging Biomarkers Alliance (QIBA)) [40] will improve liver fat quantification, MRI techniques could play a supportive role in patient management, as either an assistive tool for diagnosis or as an assessment of treatment response. Thus, to better understand the utility of these markers, either as a supporting tool at diagnosis to aid patient risk-stratification or for their utility in monitoring disease progression/regression, a long-term prospective evaluation of the changes of these quantitative markers is needed. These investigations will have the potential to yield clinically valuable information about the utility of MRI techniques in this rare orphan disease. The combination of cT1 and PDFF has also shown great utility as a marker to evaluate high-risk NASH [19]. This is particularly of interest for WD, as the wide spectrum of symptoms in patients have a considerable overlap with those seen in fatty liver disease, and the combination of markers (or their use in sequence) improves patient identification, risk stratification, and monitoring [18,41]. Thus, future studies in larger cohorts should also evaluate the potential added benefits that these non-invasive markers may have, in order to identify those who are potentially at the highest risk of disease progression. Moreover, future work should also evaluate the utility of such MRI technologies in patients with rare diseases such as cystic fibrosis, where they can potentially provide useful metrics to support disease monitoring.

## 5. Conclusions

To summarize, the diagnosis and management of Wilson’s disease can be complex due to patients’ wide spectra of symptoms; therefore, diagnosis and management of this disease can benefit from non-invasive technologies, with MR technologies showing great promise. As our hypothesis was to evaluate the potential clinical utility of MRI techniques in this rare disease, this case study shows the potential utility of quantitative multiparametric MRI metrics as adjunct biomarkers to measure disease activity and assess liver health. As one of the main challenges in organizing rare disease centres around patient numbers, prospective clinical trials should have a multi-centre design. Moreover, these studies should fully explore the utility of these promising non-invasive methods. With further validation, these technologies have the potential to provide useful information to aid clinical decision-making, such as to stratify between patients with NAFLD/NASH and those with WD.

## Figures and Tables

**Table 1:** Patient demographics and characteristics.

	Patient 1	Patient 2	Patient 3	Patient 4	Patient 5	Patient 6	Patient 7
Sex	female	female	male	male	female	female	male
Age (years)	8	11	8	10	9	16	13
BMI	13.44	15.38	16.3	18.31	17.48	26.22	23.0
**Liver enzymes**							
ALT (IU/L)	107	167	290	178	127	109	411
AST (IU/L)	54	85	117	73	84	49	398
GGT (IU/L)	35	64	87	42	34	71	204
Total Bilirubin (mg/dL)	0.31	0.71	0.32	0.36	0.53	0.41	0.2
IgG (g/L)	7.75	9.1	9.69	7.97	11.1	14.5	10.7
Gamma globulin (g/L)	11.2	16.5	13.5	11.2	17.6	15.0	10.4
Albumin (g/L)	39.3	37.6	47	42.3	46.3	35.1	42
INR	1.04	1.11	0.93	1.07	1.13	1.28	1.02
**Other markers**							
Glucose on an empty stomach (mg/dL)	72	82	87	84	106	80	95.9
Triglycerides (mg/dL)	124	134	127	159	97	76	91
Insulin on an empty stomach (mmol/L)	11.3	17.2	8.18	24.5	9.02	22.6	20.5
Leukocytes (×109/L)	7.2	9.4	5.8	5.7	4.9	4.41	6.6
Erythrocytes (×106/mcL)	4.97	4.07	4.89	5.25	4.58	4	5.16
Haemoglobin (g/dL)	13.8	12.1	13	14.9	13	13.9	14
Haematocrit (%)	41.9	37.9	39.5	44.7	39.1	41.3	40
Thrombocytes (×103/mcL)	330	406	358	394	231	243	334
Prothrombin time (seconds)	12.08	12.81	10.75	12.33	13.07	14.9	11.39
Hepatic copper content (µg/g)	426	745	873	890	539	-	1170
24 h urinary copper excretion (μg/24 h)	73	113	97	236	105	77.2	217
Serum ceruloplasmin (g/L)	0.07	0.1	0.11	0.16	0.18	0.06	0.13
**Histology scores**							
Kleiner fibrosis	2	2	1	2	2	3	3
Lobular inflammation	1	1	1	0	1	2	0
Portal inflammation	1	1	1	1	1	2	1
Steatosis	3	3	3	3	2	2	2
Ballooning	2	1	1	1	1	2	1
Cholestasis	0	0	0	0	0	0	0
Necrosis	0	0	0	0	0	1	0
Collagen proportion	10%	15%	10%	20%	15%	25%	25%

**Table 2:** Standard of care examination: clinical features, physical features, and ultrasound imaging results.

	Patient 1	Patient 2	Patient 3	Patient 4	Patient 5	Patient 6	Patient 7
**Physical examination**							
Kayser–Fleischer rings	ABSENT	PRESENT	ABSENT	ABSENT	ABSENT	PRESENT	PRESENT
PELD/MELD † score	−9	−7	−11	−9	−8	9	7
Ferenci score	7	7	8	9	6	7	11
**Genetic test**							
Mutation on ATP7B	p.His1069Gln/p.Gln1351Ter	p.His1069Gln/c.3061-2A>G	p.His1069Gln/c.3402delC	p.Leu776Pro/p.Kis1069Gln	-	p.His1069Gln/p.Gln1351c3207C>A (:)4051C>T	p.Arg969Gln/p.His1069Gln
**Ultrasound imaging and physical examination**							
Enlarged liver, spleen or kidney	ABSENT	PRESENT	ABSENT	ABSENT	ABSENT	ABSENT	ABSENT
Varices, encephalopathy or ascites	ABSENT	ABSENT	ABSENT	ABSENT	ABSENT	ABSENT	ABSENT
Alcohol use	ABSENT	ABSENT	ABSENT	ABSENT	ABSENT	ABSENT	ABSENT
Acute liver failure	ABSENT	ABSENT	ABSENT	ABSENT	ABSENT	ABSENT	ABSENT
Cholestasis	ABSENT	ABSENT	ABSENT	ABSENT	ABSENT	ABSENT	ABSENT
Pruritus	ABSENT	ABSENT	ABSENT	ABSENT	ABSENT	ABSENT	ABSENT
Uniform liver structure	PRESENT	PRESENT	PRESENT	PRESENT	PRESENT	PRESENT	PRESENT
Dilated bile ducts	ABSENT	ABSENT	ABSENT	ABSENT	ABSENT	ABSENT	ABSENT
Peritoneal fluid present	ABSENT	PRESENT	ABSENT	ABSENT	ABSENT	ABSENT	ABSENT
Fatty liver	MODERATE *	PRESENT	MODERATE *	MODERATE *	MODERATE *	MODERATE *	MODERATE *
**Additional examinations and exclusions**							
Other relevant comorbidities ^#^	ABSENT	ABSENT	ABSENT	ABSENT	ABSENT	ABSENT	ABSENT
Additional notes from physical examination	Conjunctivitis	Hepatomegaly	normal	normal	normal	normal	normal

^#^ comorbidities evaluated include: autoimmune hepatitis, primary sclerosing cholangitis, toxic liver failure, progressive familial intrahepatic cholestasis, and diabetes type 1 and type 2. † PELD scores were calculated for those < 12 years old. * MODERATE fatty liver due to slightly increased echogenicity; PRESENT fatty liver due to increased echogenicity.

**Table 3:** Quantitative multiparametric MRI and quantitative MRCP results per patient.

	Patient 1	Patient 2	Patient 3	Patient 4	Patient 5	Patient 6	Patient 7
Time between Wilson’s disease diagnosis and MRI scan (days)	693	5	1	19	2	19	7
**mpMRI**							
Fat ROI pooled mean (%)	33	24	20	17	16	10	10
cT1 whole median (ms)	990	1017	968	958	865	941	819
cT1 whole IQR (ms)	114	120	94	112	94	120	100
T2* (ms)	22.3	26.6	30.7	26.6	26.8	29.8	25.9
**MRCP+ tree metrics**							
Biliary tree volume (mL)	0.7	4.6	0.9	5.1	5.9	7.7	6.6
Gallbladder volume (mL)	11.8	21.7	15.3	31.4	66.9	61.6	8.1
Common bile duct median width (mm)	2.8	3.2	3.2	3.4	3.6	5.9	2.2
Left hepatic bile duct median width (mm)	1.9	2.1	2.6	3.5	3.6	7.3	3.3
Right hepatic bile duct median width (mm)	2.3	2.3	2.3	3.7	NA	NA	2.8
Right posteroinferior bile duct median width (mm)	NA	1.9	NA	NA	2.9	3.6	NA
Right anterior bile duct median width (mm)	NA	2.4	NA	NA	4.5	6.1	NA
Cystic duct median width (mm)	NA	2.5	NA	2.7	3.3	2	2.9
Pancreatic duct median width (mm)	1.8	2.9	NA	1.9	2.4	1.6	NA
**MRCP+ duct metrics**							
Number of ducts	8	34	10	42	40	47	63
Median duct diameter (mm)	2	2	2	2	2	2	2
Median duct length (mm)	10	15	13	11	15	16	13
Ducts with median range:							
0–1 mm	0	0	0	0	0	0	0
1–3 mm	8	33	9	38	33	43	60
3–5 mm	0	1	1	4	7	1	5
5–7 mm	0	0	0	0	0	2	0
7–9 mm	0	0	0	0	0	1	0
> 9 mm	0	0	0	0	0	0	0
Number of ducts with dilatations	0	5	0	4	5	5	6
Mean length of dilatation (mm)	0	4	0	11	5	6	6
Number of ducts with strictures	0	3	0	9	5	0	7
Mean length of strictures (mm)	0	9	0	7	7	0	11
Number of ducts with both strictures and dilatations	0	2	0	4	4	0	4

NA: was not modelled as it could not be identified from the source MRCP image.

## Data Availability

The data and analytic methods used in this study remain the property of the individual study sponsors. All deidentified participant data may be made available to other researchers upon request following permission, investigator support and following a signed data access agreement.
